# Tissue and subcellular distribution of CLIC1

**DOI:** 10.1186/1471-2121-8-8

**Published:** 2007-02-27

**Authors:** Barbara Ulmasov, Jonathan Bruno, Philip G Woost, John C Edwards

**Affiliations:** 1 Department of Internal Medicine, St. Louis University, St. Louis MO, USA; 2 UNC Kidney Center and the Division of Nephrology and Hypertension, Department of Medicine, University of North Carolina, Chapel Hill NC, USA; 3 Department of Physiology and Biophysics, Case Western Reserve University, Cleveland OH, USA

## Abstract

**Background:**

CLIC1 is a chloride channel whose cellular role remains uncertain. The distribution of CLIC1 in normal tissues is largely unknown and conflicting data have been reported regarding the cellular membrane fraction in which CLIC1 resides.

**Results:**

New antisera to CLIC1 were generated and were found to be sensitive and specific for detecting this protein. These antisera were used to investigate the distribution of CLIC1 in mouse tissue sections and three cultured cell lines. We find CLIC1 is expressed in the apical domains of several simple columnar epithelia including glandular stomach, small intestine, colon, bile ducts, pancreatic ducts, airway, and the tail of the epididymis, in addition to the previously reported renal proximal tubule. CLIC1 is expressed in a non-polarized distribution in the basal epithelial cell layer of the stratified squamous epithelium of the upper gastrointesitinal tract and the basal cells of the epididymis, and is present diffusely in skeletal muscle. Distribution of CLIC1 was examined in Panc1 cells, a relatively undifferentiated, non-polarized human cell line derived from pancreatic cancer, and T84 cells, a human colon cancer cell line which can form a polarized epithelium that is capable of regulated chloride transport. Digitonin extraction was used to distinguish membrane-inserted CLIC1 from the soluble cytoplasmic form of the protein. We find that digitonin-resistant CLIC1 is primarily present in the plasma membrane of Panc1 cells. In T84 cells, we find digitonin-resistant CLIC1 is present in an intracellular compartment which is concentrated immediately below the apical plasma membrane and the extent of apical polarization is enhanced with forskolin, which activates transepithelial chloride transport and apical membrane traffic in these cells. The sub-apical CLIC1 compartment was further characterized in a well-differentiated mouse renal proximal tubule cell line. The distribution of CLIC1 was found to overlap that of megalin and the sodium-phosphate cotransporter, NaPi-II, which are markers of the apical endocytic/recycling compartment in proximal tubule.

**Conclusion:**

The cell and tissue specific patterns of CLIC1 expression suggest it may play distinct roles in different cell types. In certain polarized columnar epithelia, it may play a role in apical membrane recycling.

## Background

A variety of distinct chloride channel activities have been described that carry out many essential roles in organismal physiology [1]. Disruption or disregulation of chloride conductances are central to several well-described disease processes, perhaps most notably, cystic fibrosis [2]. Although the proteins responsible for some chloride conductances have been described, there are still multiple physiologically important chloride channel activities for which the molecular basis is not yet understood. Identification of the proteins responsible for these "orphan" channel activities will be an important step in more fully understanding ion transport physiology in health and disease.

CLICs are a closely related family of chloride channel proteins [3,4]. In mammals, the family consists of six genes named CLICs one through six. The CLIC family is defined by a conserved, approximately 230 amino acid core sequence which comprises the C-termini of all known CLICs. Sequences amino-terminal to the core region are divergent both in sequence and in size. The highly conserved CLIC domain has no homology to other known chloride channel proteins; however it does show low but significant homology to the family of glutathione-S-transferases [5].

Evidence that CLICs are chloride channels comes from a variety of experiments and systems. Exogenous expression of CLIC1, 3, 4, and 5 have all been reported to result in the appearance of novel chloride channel activity using a variety of expression and assay systems [6-11]. Conclusive proof that a purified CLIC can function as a channel was first presented for CLIC1. Expression of CLIC1 in bacteria followed by purification to apparent homogeneity was shown to yield a distinct channel activity when reconstituted in phospholipid membranes, demonstrating unequivocally that CLIC1 alone, without any other subunits or regulatory proteins, functions as an anion-selective channel [12].

CLIC proteins have biochemical properties which are quite unusual for ion channels. Unlike most other ion channels, CLIC proteins exist in cells both as integral membrane proteins and as soluble, cytoplasmic proteins. *In vitro*, soluble aqueous CLIC1 has been shown to insert directly into pre-formed phospholipid membranes, with this spontaneous insertion leading to active channels [13-16].

To date, papers from three independent research groups have reported observations of channel activity associated with purified recombinant CLIC1 [12-16]. While each group finds channels that can be inhibited by IAA-94, there is little consensus regarding single channel properties including single channel conductance, ion selectivity, lipid dependence, and effects of oxidation. In particular, Tulk *et al*. [12,13] report a single channel conductance of more than twice the magnitude of that reported by others [14-16]. Furthermore, estimates of the ratio of chloride to potassium permeabilities ranges from very high [14] through intermediate [12,13] to very low [16]. Each group used somewhat different methods, but the basis for these discrepancies in observed fundamental properties of the protein remains unexplained.

Although CLIC1 clearly can function as a channel *in vitro*, the role of CLIC1 in normal physiology remains uncertain and the membrane fraction in which it primarily resides has not been clearly identified. Based on a staining pattern in CHO cells, it was initially proposed that CLIC1 is a channel of the nuclear membrane [10]. Our initial studies in cultured human cells revealed a pattern of punctate staining throughout the cytoplasm with little nuclear localization, leading us to propose that CLIC1 could be a channel of intracellular cytoplasmic organelles [17]. Subsequently, single channels consistent with CLIC1 have been reported on the plasma membrane of cells and the correlation of expression with cell cycle stage has led to the proposal that CLIC1 may be involved in cell cycle regulation [18]. One significant problem confounding prior studies of subcellular distribution of CLIC1 is the observation that CLICs are present in cells both as a membrane-inserted, integral membrane protein, and as a soluble, apparently cytoplasmic protein. In some cell types, the soluble CLIC1 is significantly more abundant than the membrane-associated form. Thus, cell staining techniques which do not distinguish between soluble and membrane-associated CLIC may lead to erroneous conclusions.

An important clue to the role of a protein is its distribution in normal cells and tissues. CLIC1 had previously been shown to be abundant in apical membranes of renal proximal tubule cells [17], in a cytoplasmic distribution in placental trophoblast epithelium [19], and in the acrosomal region of mature spermatozoa [20]. In order to address this question further, we have explored the distribution of expression of endogenous CLIC1 in a variety of mouse tissues using immunohistochemical techniques. In this paper, we show that CLIC1 is expressed in the apical domain of a variety of simple columnar epithelia as well as in a non-polarized distribution in the basal cell layer of some stratified squamous epithelia, and is present diffusely throughout skeletal muscle fibers. We further investigated subcellular localization of CLIC1 in three distinct cell lines using dual-label confocal immunofluorescence microscopy. To distinguish between membrane associated and cytoplasmic CLIC1, we used digitonin extraction prior to fixation to selectively solubilize cytoplasmic proteins [21-23]. In Panc1 cells, a relatively undifferentiated non-polarized cell line which expresses abundant CLIC1, we find that the characteristic intracellular punctate staining pattern previously reported is due to digitonin-extractable, presumably cytoplasmic, CLIC1, while the majority of the digitonin-resistant CLIC1 colocalizes with plasma membrane markers. In T84 cells, a highly polarized colonic epithelial cell line, CLIC1 expression is polarized to the apical domain of the cell, reproducing the polarized expression observed in normal tissues. The extent of apical polarization is enhanced in response to stimulation of the cells with forskolin, which activates transepithelial chloride transport in these cells via a CFTR-mediated mechanism [24]. Unlike in Panc1 cells, the pattern of distribution in T84 cells is not significantly altered by extraction with digitonin. Double label experiments indicate that the mass of the digitonin-resistant CLIC1 does not precisely colocalize with the apical plasma membrane but is immediately below it. In MPTC, an immortalized mouse kidney proximal tubule cell line, the distribution of CLIC1 intersects with that of both megalin and NaPi2, markers of the apical membrane endocytic/recycling compartment [25,26].

We conclude that CLIC1 is widely expressed in a specific set of epithelial and non-epithelial cell types where it shows tissue- and cell-specific patterns of subcellular localization. The data suggest CLIC1 may play different roles in various cells, one of which may be to contribute to endocytosis and/or recycling of the apical plasma membrane in certain polarized epithelial cells.

## Results

### Characterization of antibodies raised against CLIC1

**Figure 1:**
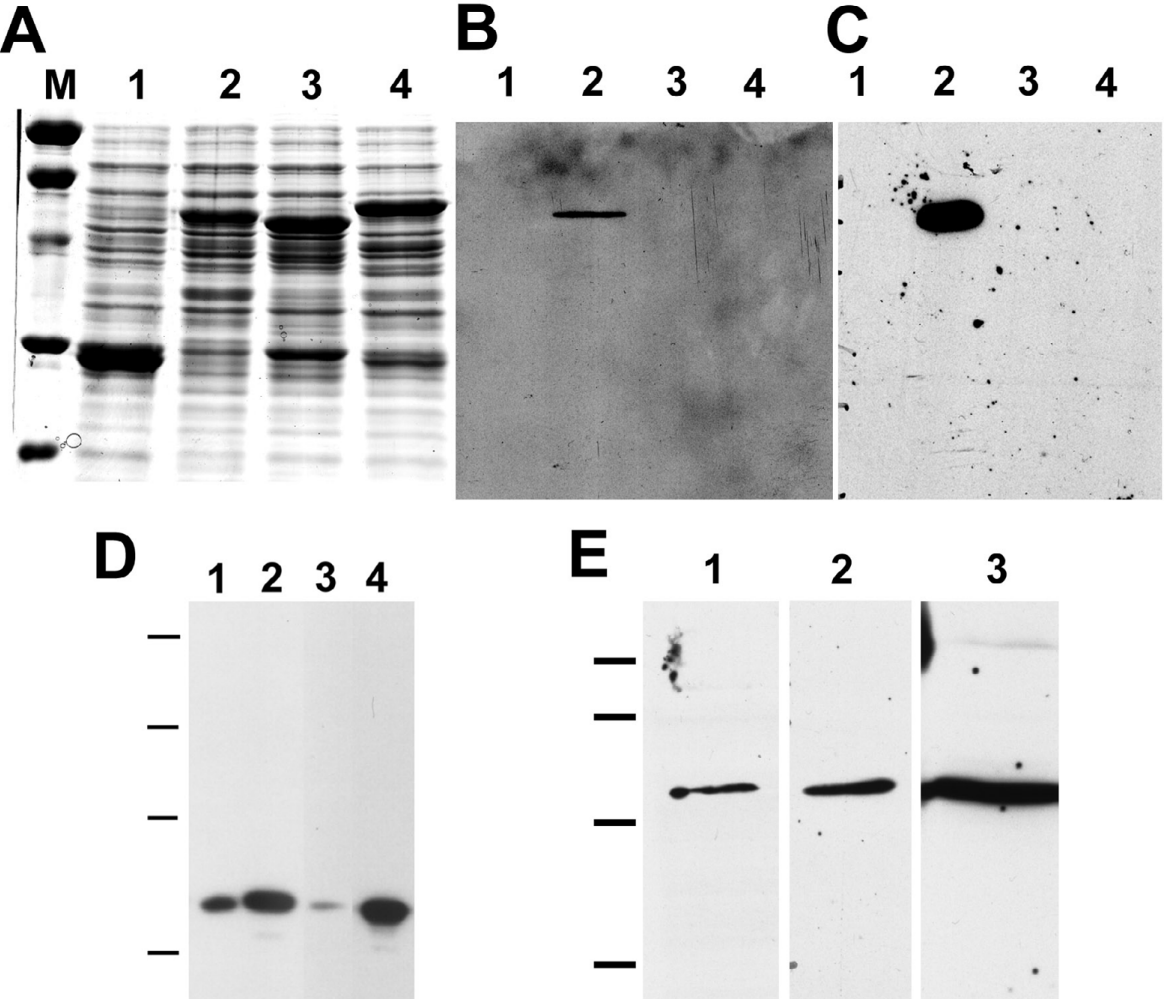
**Characterization of antibodies**. **A. **Lysates of bacteria expressing glutathione-S-transferase (GST, lane 1) or GST-CLIC1 (lane 2), GST-CLIC4 (lane 3) or GST-CLIC5 (lane 4) fusion proteins were separated by SDS-PAGE and stained with Coomassie blue. Lane M contains molecular mass standards of 96, 66, 45, 31, and 21 kDa. **B. **Gel identical to that shown in panel A, blotted and probed with AP1089. **C. **Gel as in B, probed with 9F5. **D. **Mouse kidney microsomal membranes (lane 1) or whole cell lysates from Panc1 cells (lane 2), HELA cells (lane 3) or HELA cells overexpressing exogenous CLIC1 (lane 4) were separated on a 10% SDS-polyacrylamide gel, blotted, and probed with AP1089. Sample lanes each contained 30 μg of protein. Migration positions of molecular mass standards of 96, 66, 45, and 31 kDa are indicated. **E. **Whole cell lysates from Panc1 cells (lane 1), T84 cells (lane 2) or mouse kidney microsomal membranes (lane 3) were separated on 12% SDS-polyacrylamide gels, blotted, and probed with 9F5 monoclonal antibody. Lanes contain 5 μg of each protein sample. Migration positions of molecular mass standards of 66, 45, 31, and 21 kDa are indicated.

**Figure 4:**
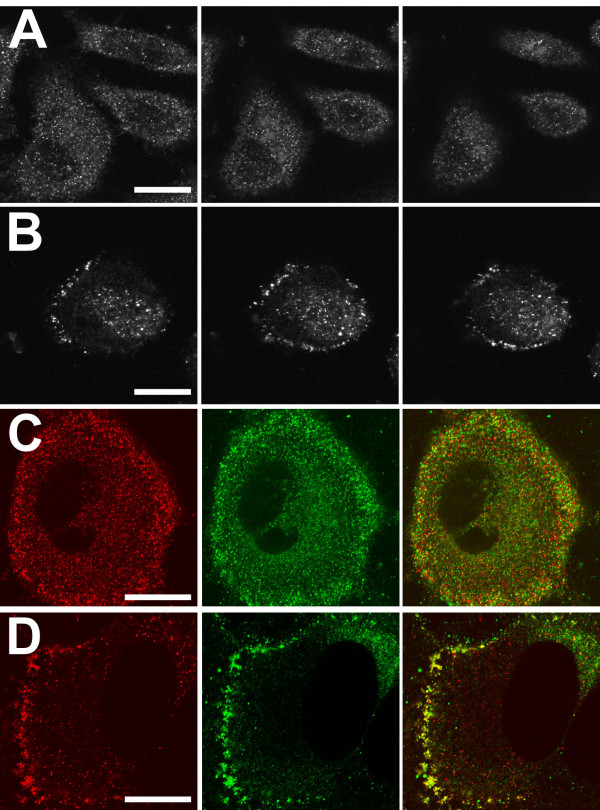
**Effect of digitonin extraction on distribution of CLIC1 in Panc1 cells**. Panc1 cells were fixed with PLP without (**A, C**) or with (**B, D**) prior extraction with digitonin. Cells were then stained with AP1089 (**A, B**) or with both AP1089 and 9F5 (**C, D**) and imaged using confocal microscopy. In A and B, images are shown from the very base of the cell (left panel) plus images at focal planes 2 (center) and 4 (right) μm higher. Collection of images in B required higher sensitivity than A. Parallel cultures stained with control antisera and imaged under identical conditions were blank (not shown). In panels C and D, cells were double stained with AP1089 with Alexafluor565-conjugated goat anti-rabbit IgG (red, left panel) and 9F5 with Alexafluor488-conjugated goat anti-mouse IgG (green, center panel) without (**C**) or following (**D**) digitonin extraction. A merged image for each pair is shown on the right. Scale bar in A and B represent 25 μm, scale bars in C and D represent 20 μm.

The affinity-purified 1089 antisera, AP1089, and the mouse monoclonal antibody, 9F5, were used to probe western blots of bacterial cell lysates expressing glutathione-S-transferase (GST) or GST fusion proteins containing full length human CLIC1, CLIC4, and CLIC5A (Fig. 1A–C). A fusion protein of the predicted size is the most abundant protein in each lane and is easily seen on a Coomassie Blue stained gel (Fig. 1A). Both AP1089 (Fig. 1B) and 9F5 (Fig. 1C) only recognize the CLIC1 fusion protein. In protein preparations from mouse kidney, Panc1 cells or HELA cells, AP1089 recognizes a single band with apparent molecular weight of 34,000 (Fig. 1D, lanes 1–3). Overexpression of human CLIC1 in HELA cells using a vaccinia-driven expression system leads to this band becoming more prominent without the appearance of any other band (Fig. 1D, lane 4). Similarly, the monoclonal antibody 9F5 recognizes a single protein with apparent molecular weight of 34,000 in Panc1, T84, and mouse kidney proteins (Figure 1E). Of note, both CLIC4 (running at about 31,000 molecular weight) and CLIC5B (running at about 65,000 molecular weight) are expected to be present in mouse kidney and are not recognized by the antibody while CLIC1 is robustly detected. Finally, AP1089 and 9F5 give identical patterns when used to stain Panc1 cells either without or with digitonin extraction (see figure 4 below). Thus, we conclude that both AP1089 and monoclonal 9F5 are specific for CLIC1.

### Immunolocalization of CLIC-1 in mouse tissues

**Figure 2:**
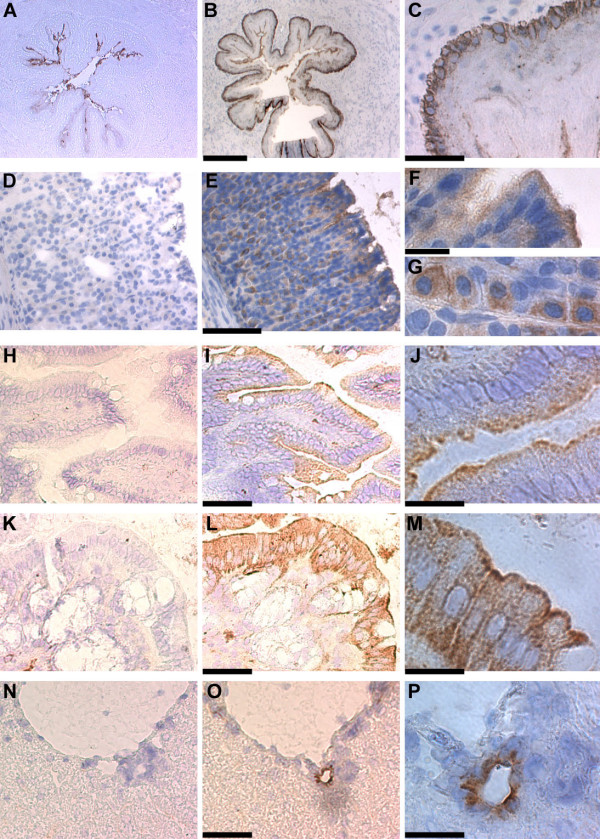
**Staining of mouse frozen tissue sections for CLIC1**. Each row displays images from a single tissue. The left column contains sections stained with control antibody, the center column contains matched sections stained with AP1089. Scale bars displayed with the center image apply to both the left and center images of that row. The right column presents a high power view of the corresponding tissue stained with AP1089. **A, B. **Esophagus imaged with 4× objective. Bar = 200 μm. **C. **Esophagus with 20× objective showing basal epithelial cell layer. Bar = 50 μm. **D, E. **Glandular stomach with 10× objective. Bar = 100 μm. **F. **Surface epithelium of glandular stomach with 40× objective. **G. **Subepithelial glands of glandular stomach with 40× objective. Bar = 20 μm, applies to both panel F and G. **H, I. **Small intestine (jejunum) with 20× objective. Bar = 50 μm. **J. **Small intestine with 60× objective. Bar = 20 μm. **K, L: **Colon with 20× objective. Bar = 50 μm. **M. **Colon with 60× objective. Bar = 20 μm. **N, O. **Liver with 20× objective. Bar = 50 μm. **P. **Liver with 60× objective, bar = 20 μm.

**Figure 3:**
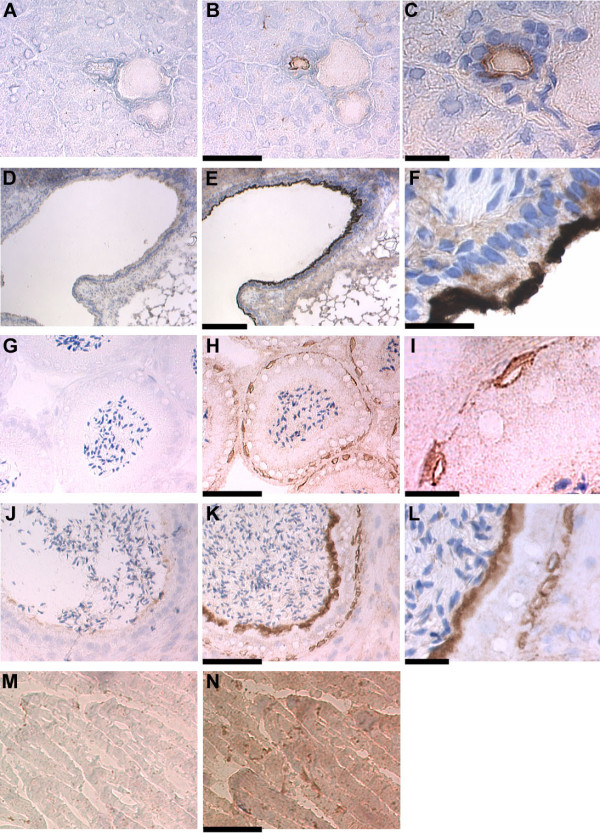
**Staining of mouse frozen tissue sections for CLIC1**. Images are arranged as in figure 2. **A, B. **Pancreas with 10× objective. Bar = 100 μm. **C. **Pancreatic duct from B with 40× objective. Bar = 20 μm. **D, E. **Lung with 4× objective. Bar = 200 μm. **F. **Airway epithelium as in E with 60× objective. Bar = 20 μm. **G, H. **Head of epididymis with 10× objective. Bar = 100 μm. **I. **Epithelial layer from head of epididymis as in H with 60× objective. Bar = 20 μm. **J, K. **Tail of epididymis with 10× objective. Bar = 100 μm. **L. **Tail of epididymis as in K with 40× objective. Bar = 20 μm. **M, N. **Skeletal muscle under 20× objective. Bar = 50 μm.

Mouse esophagus, forestomach, glandular stomach, small intestine, colon, submandibular gland, lung, liver, gall bladder, spleen, pancreas, heart, skeletal muscle, skin, adrenal, testis, and epididymis were each stained with AP1089. Representative images from tissues in which staining was observed are shown in figures 2 and 3. In each figure, the left column presents a low power view of a section stained with the control antibody and the middle column presents a comparable image from a section stained with AP1089. The right column is a high power view of the same tissue. The horseradish peroxidase reaction product indicating antibody binding is brown while the hematoxalyin counterstain, primarily labeling nuclei, is blue.

The esophagus and forestomach of the mouse are lined by a stratified squamous epithelium surrounded by smooth muscle. Images of esophageal sections are shown in figure 2A–C. Low power views of sections stained with control antibody (Fig. 2A) or with AP1089 (Fig. 2B) show intense CLIC1 staining of the basal epithelial layer. In addition, there is background reaction product associated with the most superficial layer of squamous epithelial cells which is no different between control and CLIC1-stained section. A higher power view is presented in figure 2C, showing the intensely stained basal layer separating the underlying smooth muscle in the upper left from the stratified squamous epithelial layers on the lower right. The pattern of forestomach staining was identical (not shown).

Glandular stomach is lined with a simple columnar epithelium. The surface epithelium is composed of a uniform layer of mucous-secreting cells while the underlying glands consist of a mixture of enzyme-secreting chief cells, acid-secreting parietal cells, mucin-secreting goblet cells, and gastrin-secreting G cells. Staining of glandular stomach is shown in figures 2D–G. Low power images (Fig. 2D–E) show clear staining of both surface and glandular epithelial cells above background without appreciable staining of underlying connective tissue or muscle. A higher power view of surface epithelium (Fig. 2F) shows homogeneous staining of all the cells of the epithelium with marked apical polarization. A higher power view of glands (Fig. 2G) reveals a quite different staining pattern with homogeneous staining throughout the cytoplasm of a subset of the gland cells.

Images of small intestine (Fig. 2H–J) and colon (Fig. 2K–M) show selective staining of the simple columnar epithelium with marked apical polarization and without appreciable staining of underlying connective tissue, muscle, or, in the colon, subepithelial glands.

Staining of liver is shown in figures 2N–P. Low power images in 2N-O show a central vein at the top with a intrahepatic bile duct immediately below it, surrounded by hepatocytes. The hepatocytes show no detectable staining, but there is clear staining of intrahepatic bile ducts. 2P is a higher power image of the bile duct shown in 2O. The staining is concentrated in the apical pole of the bile duct epithelial cells. The gall bladder epithelium, like the intrahepatic bile ducts, stained for CLIC1 in an apical distribution (not shown).

The pancreas showed intense staining of the branches of the pancreatic ducts with no apparent staining of the exocrine acini or other structures (Fig. 3A–C). Low power images in 3A-B show a vein, artery, and pancreatic duct surrounded by pancreatic acini. A higher power image of the duct (Fig. 3C) reveals that CLIC1 is most prominent in the apical pole of the ductal epithelium. The islets do not appreciably stain for CLIC1 (not shown).

Low power images of the lung (Fig. 3D–E) show an airway on the left with alveoli on the right. There is intense staining for CLIC1 in the airway epithelium with no appreciable staining of alveoli or vascular structures in the lung. A high power image of the airway epithelium (Fig. 3F) shows marked apical polarization of CLIC1 staining.

The epididymal epithelium is a transitional epithelium with both basal cells along the basement membrane and columnar epithelial cells lining the lumen. There are structural and functional differences between the head and the tail portions of the organ, and these two portions showed distinct patterns of CLIC1 staining. Images from the head of the epididymis (Fig. 3G–I) show sperm within the lumen surrounded by the epithelium consisting of tall columnar cells and underlying basal cells. Only the basal epithelial cells stain for CLIC1. A high power image (Fig. 3I) shows stained basal cells underlying columnar cells to the lower right. As in the upper GI tract, the staining of the basal cells does not appear to be polarized. In the tail (Fig. 3J–L) both the basal cells and the columnar epithelial cells stain intensely with the distribution of CLIC1 in the columnar epithelial cells showing a marked apical polarization of distribution. A high power image from the tail of the epididymis (Fig. 3L) shows staining of both basal cells to the right, and the apical domain of columnar cells which lies immediately below the luminal sperm on the left.

Skeletal muscle showed clear staining for CLIC1 above control (Fig. 3M–N). The signal is diffusely distributed throughout the skeletal muscle cells without apparent specific localization.

Submandibular gland, heart, spleen, skin, adrenal gland, and testis all showed no staining with AP1089 (not shown).

### Distribution of CLIC1 in Panc1 cells

Panc1 cells were grown on cover slips, fixed, permeabilized and stained for CLIC1. Cells fixed directly with PLP and stained with the AP1089 antibody (Fig. 4A) showed the typical intracellular punctuate staining pattern that we had reported previously using a different CLIC1 antibody [15]. There is negative staining of the nucleus in most cells, indicating that staining of the cytoplasm is more intense than staining of the nucleus. To characterize the staining patterns further, cells were fixed, permeabilized, and stained simultaneously with both the polyclonal rabbit AP1089 and the monoclonal mouse 9F5 (Fig. 4C). The two antibodies give essential identical staining patterns with nearly perfect colocalization. Double staining for CLIC1 with AP1089 and with monoclonal antibodies specific for markers of the endoplasmic reticulum (BiP), the Golgi apparatus (Golgi 58 K protein), lysosomes (Lamp1), and trans-Golgi network (TGN46) all failed to show any significant colocalization (not shown).

**Figure 5:**
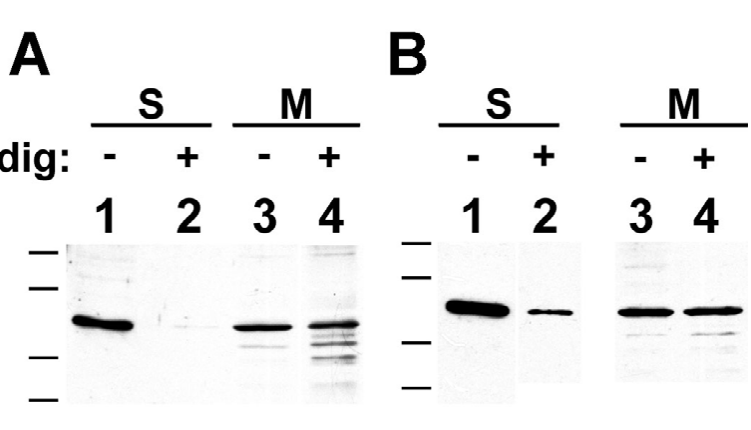
**Digitonin extraction selectively removes soluble CLIC1 from Panc1 and T84 cells**. Panc1 cells (**A**) or T84 cells (**B**) were grown to confluence. Parallel cultures were untreated (lanes 1 and 3) or extracted with digitonin (lanes 2 and 4) prior to homogenization and preparation of soluble (S) and membrane (M) fractions. Two micrograms of Panc1 soluble protein (A, lanes 1, 2), 8.5 μg of Panc1 membrane protein (A, lanes 3, 4), 2 μg of T84 soluble protein (B, lanes 1, 2), or 10 μg of T84 membrane protein (B, lanes 3, 4) were separated on SDS-PAGE, blotted and probed with AP1089 antibody.

The staining pattern of CLIC1 must result from superimposed distribution of the soluble and membrane-inserted fractions of the protein. We were concerned that the distribution of the abundant cytoplasmic CLIC1 might be obscuring the distribution of the less abundant membrane-inserted form of the protein. The non-ionic detergent, digitonin, has been reported to solubilize selectively cytoplasmic proteins while leaving membrane-inserted proteins in place [21-23]. To determine whether we could use digitonin to remove selectively non-membrane-associated CLIC1 from cells, Panc1 cells were grown on plastic culture dishes and extracted with 0.004% (w/v) digitonin at room temperature for two minutes. Soluble and membrane fractions were then prepared from the cells by centrifugation. Two micrograms of soluble protein or 8.5 μg of membrane protein were blotted and probed for CLIC1 (Fig. 5A). Clearly, digitonin extraction removes the soluble CLIC1 from these cells without significantly removing the membrane-associated CLIC1.

Since digitonin selectively removes soluble CLIC1 from cultured cells, digitonin extraction prior to fixation and immunostaining should reveal the true subcellular distribution of membrane-inserted CLIC1. Panc1 cells on coverslips were subjected to a two minute extraction with 0.004% (w/v) digitonin prior to fixation with PLP and staining for CLIC1 (Fig. 4B). The resulting staining pattern is weaker than with non-digitonin extracted cells, requiring more sensitive detection settings to obtain an image. However, the staining pattern is markedly greater than and different from matched preparations stained with control antibody (not shown). After digitonin extraction, the prominent intracellular punctate staining pattern has disappeared, leaving a fainter wispy peripheral localization of the residual staining, plus some staining of a circular structure suggestive of the nucleus. Digitonin-extracted cells were also double stained with both anti-CLIC1 antibodies (Fig. 4D). Both the polyclonal rabbit and monoclonal mouse antibodies give essentially identical staining patterns with loss of the intracellular punctuate pattern leaving peripheral staining at the edge of the cells.

**Figure 6:**
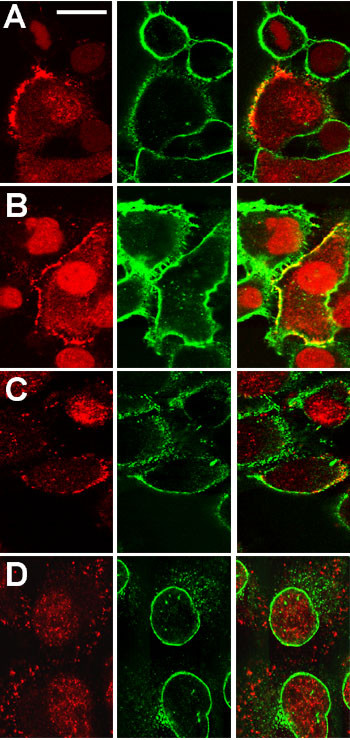
**Colocalization of CLIC1 with markers of plasma membrane and nucleus following digitonin extraction**. Panc1 cells were grown on glass coverslips and extracted with digitonin prior to PLP fixation and staining for CLIC1 (red) and subcellular compartment markers (green). The right column in each set is a merged image generated from the red (left column) and green (center column) channels. **A, B. **Two separate fields stained for CLIC1 (red) and Integrin α2 (green), a plasma membrane marker. **C. **Cells stained for CLIC1 (red) and Annexin2 (green), a plasma membrane marker. **D. **Cells stained for CLIC1 (red) and Nucleoporin p62 (green) a marker of nuclear envelope. The scale bar in panel A represents 20 μm and applies to all panels.

Double staining for CLIC1 with AP1089 and with monoclonal antibodies specific for markers of the plasma membrane or the nuclear membrane of Panc1 cells that had been extracted with 0.004% (w/v) digitonin are shown in figure 6. The peripheral stain clearly colocalizes with the plasma membrane markers Integrin α2 (Fig. 6A, B) and Annexin II (Fig. 6C), with the intensity and extent of plasma membrane CLIC1 staining varying significantly from cell to cell and between different regions within individual cells. The nuclear envelope marker, Nucleoporin p62 (Fig. 6D), confirms that there is residual staining of CLIC1 within the nucleus following digitonin extraction and this staining is distinct from the nuclear envelope itself.

**Figure 7:**
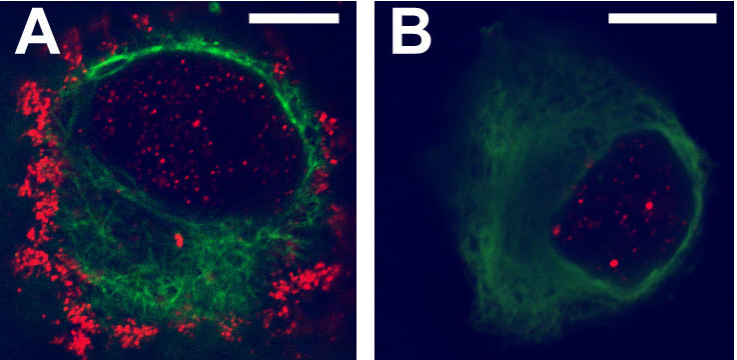
**Differential extraction of CLIC1 from Panc1 Cells with digitonin and Triton X-100**. Panc1 cells grown on glass coverslips were extracted with 0.004% (w/v) digitonin (**A**) or 0.5% (v/v) Triton X-100 (**B**) prior to fixation and staining for cytokeratin (green) and CLIC1 (red). The scale bar in each panel represents 10 μm.

The behavior of cytoskeletal proteins during digitonin extraction has not been well described, but one might expect cytoskeleton to be left largely intact by a mild detergent like digitonin. Thus digitonin-resistant CLIC1 could conceivably represent cytoskeletal rather than membrane distribution. In contrast to digitonin, Triton X-100 has been reported to solubilize both cytoplasmic and membrane protein while leaving the cytoskeleton intact [11,27]. To distinguish whether the digitonin-resistant CLIC1 represents membrane or cytoskeletal association, we compared the distribution of CLIC1 in cells which had been extracted with digitonin with that in cells which had been extracted with Triton X-100. Panc1 cells were grown on coverslips and subjected to a two minute extraction with 0.004% (w/v) digitonin or a five minute extraction with 0.5% (v/v) Triton X-100. The cells were then fixed and processed as usual and stained with the AP1089 antibody for CLIC1 and with a monoclonal pan-anti-cytokeratin antibody to label the intermediate-filament cytoskeleton. Results are shown in figure 7. Clearly both the digitonin and Triton X-100 extractions leave the cytoskeleton intact while the Triton X-100 extraction completely eliminates the peripheral plasma membrane CLIC1 staining seen in the digitonin extracted cells. We conclude that the peripheral CLIC1 staining represents CLIC1 in the plasma membrane and not associated with the cytoskeleton. Of note, some of the punctuate intranuclear staining is Triton X-100 resistant, suggesting it may be associated with nuclear skeletal elements.

### Distribution of CLIC1 in T84 cells

**Figure 8:**
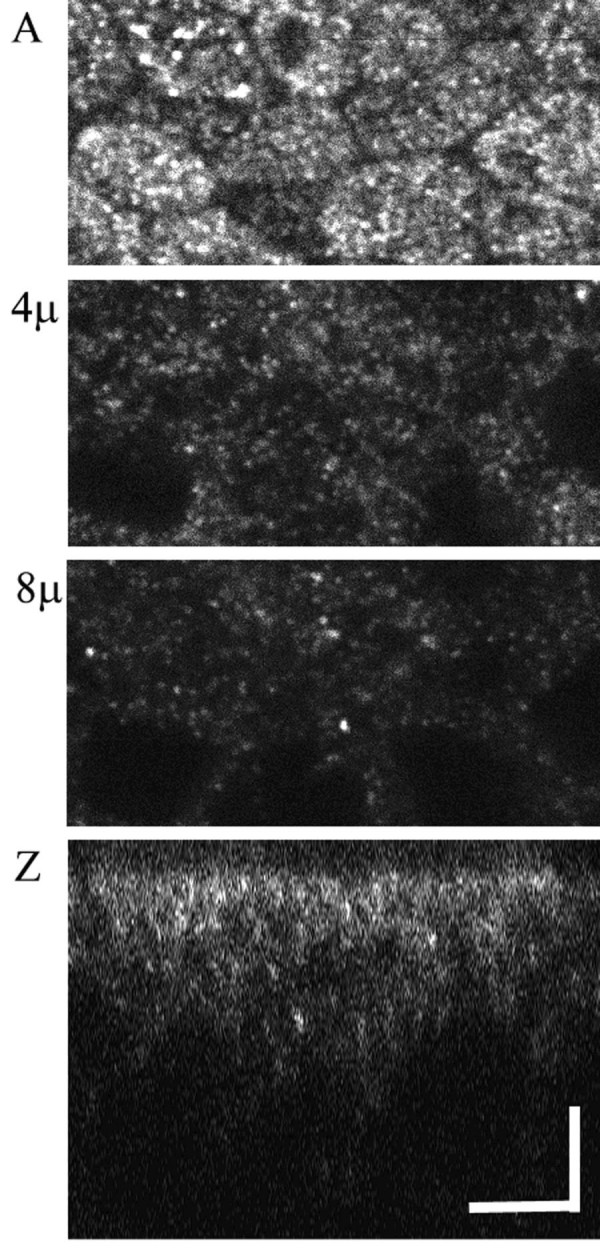
**CLIC1 in T84 cells**. Cells were grown to confluence on permeable supports, fixed, stained for CLIC1, and a stack of Z images at 0.2 μm intervals collected by confocal microscopy. **A: **image from the apex of the cells. **4μ: **image taken 4 μm below image A. **8μ: **image take 8 μm below image A. **Z: **vertical section generated from a 2 μm thick slice through the center of the stack of images. The scale bars represent 5 μm.

We also examined the distribution of CLIC1 in T84 cells, a well-differentiated human colon cancer cell line. On permeable supports, T84 cells form a columnar epithelium which demonstrates CFTR-dependent, cAMP-activated chloride secretion (24). A T84 monolayer was stained with AP1089. Confocal images from several focal planes are shown in figure 8 as well as a vertical section created from a narrow slice through the stack of horizontal images. CLIC1 stains punctate structures which are most abundant in the apical pole of the cell. As with the Panc1 cells, the CLIC1 in T84 cells failed to colocalize with markers of endoplasmic reticulum, Golgi, lysosomes, or trans-Golgi network (not shown).

**Figure 9:**
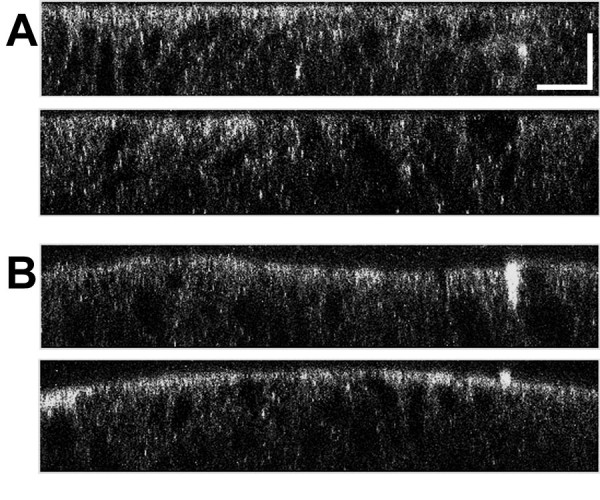
**Redistribution of CLIC1 in T84 cells in response to forskolin**. Confluent monolayers of T84 cells grown on filters were fixed directly (**A**) or pretreated with 10 μM forskolin in growth medium for 10 minutes prior to fixation (**B**). Cells were stained for CLIC1 and Z-section images created from 2 μm thick slices taken through stacks of confocal images taken at 0.2 μm intervals. Two representative Z-sections are shown from each specimen. The culture surface is at the bottom of each image, the apical surface of the cells at the top. Scale bar represents 10 μm.

To determine whether activation of chloride transport alters the distribution of CLIC1, cell monolayers were fixed and imaged either in a control state or after stimulation with forskolin to increase intracellular cAMP. Reconstructed vertical sections through representative regions are shown in figure 9. The apical polarization of CLIC1 distribution is clearly enhanced following forskolin treatment.

As with the Panc1 cells, we wished to distinguish the distribution of membrane-inserted CLIC1 from the soluble cytoplasmic fraction of the protein. To determine whether digitonin extraction would remove soluble CLIC1 from T84 cells, T84 monolayers were extracted with 0.004% (w/v) digitonin, fractionated into soluble and membrane fractions, and probed for CLIC1 as described above. As shown in figure 5B, digitonin extraction greatly reduced the soluble CLIC1 without appreciably changing membrane-associated CLIC1. The removal of soluble CLIC1 was not as complete with T84 cells as with Panc1, but a large majority of the soluble CLIC1 was clearly removed.

**Figure 10:**
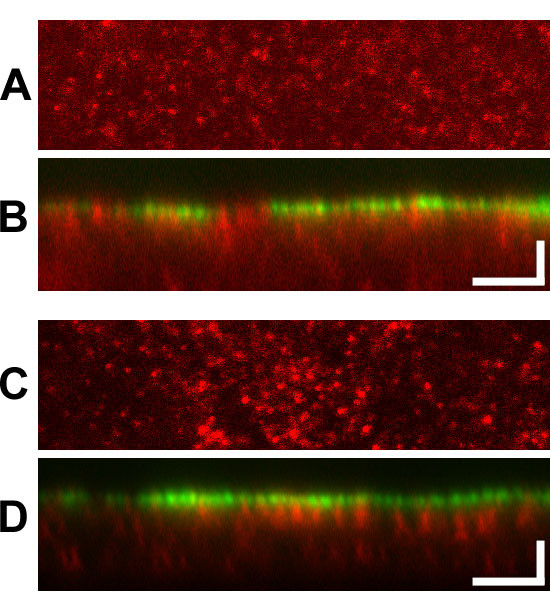
**Effect of digitonin extraction on distribution of CLIC1 in T84 cells**. Confluent T84 cell monolayers were stained with FITC-wheat germ agglutinin on ice, then either fixed directly (**A, B**) or following digitonin extraction (**C, D**). Cells were then stained for CLIC1 and confocal images obtained at 0.3 μm intervals. For both control (**A, B**) and digitonin extracted (**C, D**) samples, a red channel image from the focal plain with the most intense CLIC1 staining (panes A and C) and a merged Z-section generated from a 3.5 μm thick slice through the center of the stack of images (panes B and D) are shown.

We then compared the staining pattern of CLIC1 in T84 cells with or without extraction with 0.004% (w/v) digitonin. To mark the apical plasma membrane, the cells were labeled with FITC-conjugated wheat germ agglutinin (WGA) prior to permeabilization and staining for CLIC1. A stack of images at 0.3 μm steps were taken through each cell monolayer. Results are shown in figure 10. Two images from a representative field are shown from both the control (Fig. 10A, B) and digitonin-extracted (Fig. 10C, D) cultures. The upper image of each pair is an *en face *view of the CLIC1 distribution in the focal plane with the most intense CLIC1 staining (Fig. 10A, C), which for both samples was near the apical surface of the cell monolayer. The lower image of each pair is a Z-section generated from the stack of images, showing the distribution of both CLIC1 (red) and the apical membrane marker FITC-WGA (green). Following digitonin extraction, overall staining intensity for CLIC1 was lower, but the distribution did not appear remarkably different from that of unextracted cells. CLIC1 was still present in punctate structures that were more abundant in the apical pole of the cell. Digitonin extraction did appear to decrease the diffuse faint staining between the more intensely stained discreet structures. Although there was clearly some colocalization, the level of the most intense staining for CLIC1 either with or without digitonin extraction was slightly below the most intense staining level for WGA, suggesting that the bulk of the digitonin-insoluble CLIC1 is not in the plasma membrane itself, but congregated immediately below it.

**Figure 11:**
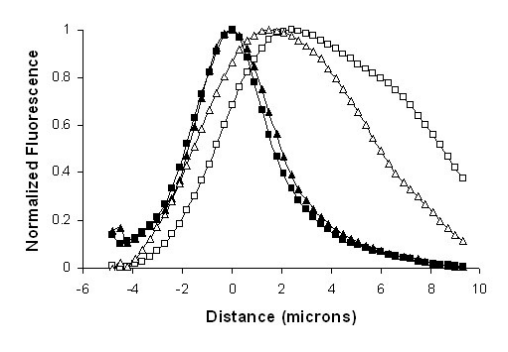
**Redistribution of digitonin-resistant CLIC1 in T84 Cells in response to forskolin**. Normalized fluorescence intensity for FITC-WGA (closed symbols) or CLIC1 (open symbols) in control T84 cell monolayers (squares) or forskolin-treated monolayers (triangles), derived from stacks of confocal images and plotted as a function of the vertical distance from the peak of WGA. Data is averaged from three separate stacks derived from each monolayer.

We examined the distribution of digitonin-insoluble CLIC1 in response to forskolin. Confluent T84 cell monolayers were exposed to HEPES-buffered saline (HBS), or HBS with 10 μM forskolin for 10 minutes at 37°C, then chilled and their apical surface labeled with FITC-WGA. The cells were then subjected to extraction with 0.004% (w/v) digitonin followed by fixation, permeabilization and staining for CLIC1. Stacks of confocal images covering square 36 × 36 μm fields were collected at 0.3 μm vertical intervals. Fluorescence intensity in each channel was summed from each focal plane. The peak of intensity of the FITC-WGA staining was taken as the apical surface of the cell and the stacks of images were aligned to this reference point. Intensities were averaged from aligned stacks of images collected from 3 separate fields from each cell monolayer. The fluorescence intensities were normalized to the maximum and minimum recorded intensity in each channel and were plotted as a function of distance in the vertical dimension as shown in figure 11. In both control and forskolin-treated cells, the WGA distribution is sharp, indicating the apical membrane lies within a narrow band in these image stacks. In the control cell layer, the peak of the CLIC1 staining lies 2.4 μm below the peak of WGA staining and the distribution of CLIC1 appears rather broad. Following forskolin treatment, the peak CLIC1 staining lies 1.5 μm below the peak WGA stain and the distribution of the CLIC1 is narrower.

These results indicate that CLIC1 in T84 cells resides in a membrane compartment lying immediately below the apical plasma membrane and that forskolin causes the distribution of membrane-inserted CLIC1 to become more apically polarized. However, even after forskolin, the bulk of the CLIC1 is not in the plasma membrane itself but is immediately below it.

### Distribution of CLIC1 in immortalized mouse proximal tubule cells

**Figure 12:**
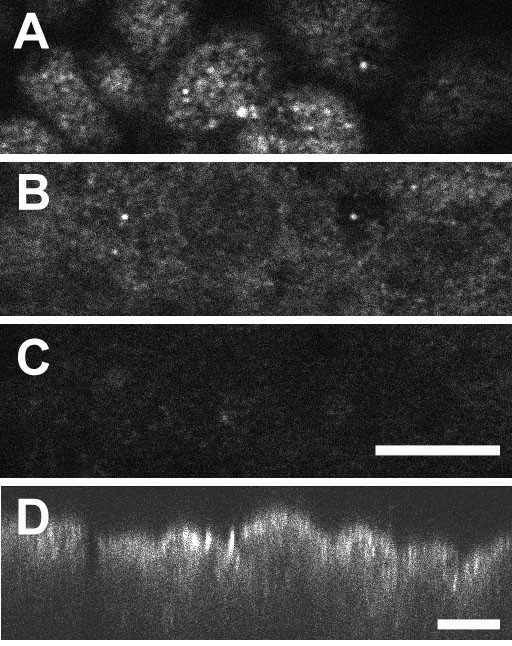
**CLIC1 in MPTC cells**. MPTC cell line was grown on collagen-coated filters, fixed, and stained with AP1089. Confocal images from the apical pole of the cell (**A**) and 5 microns (**B**) or 10 microns (**C**) below are shown. Scale bar represents 10 microns. **D. **Z section taken from a separate field along a single pixel line using 153 planes of focus covering 24 microns in depth. Scale bar represents 10 microns.

**Figure 13:**
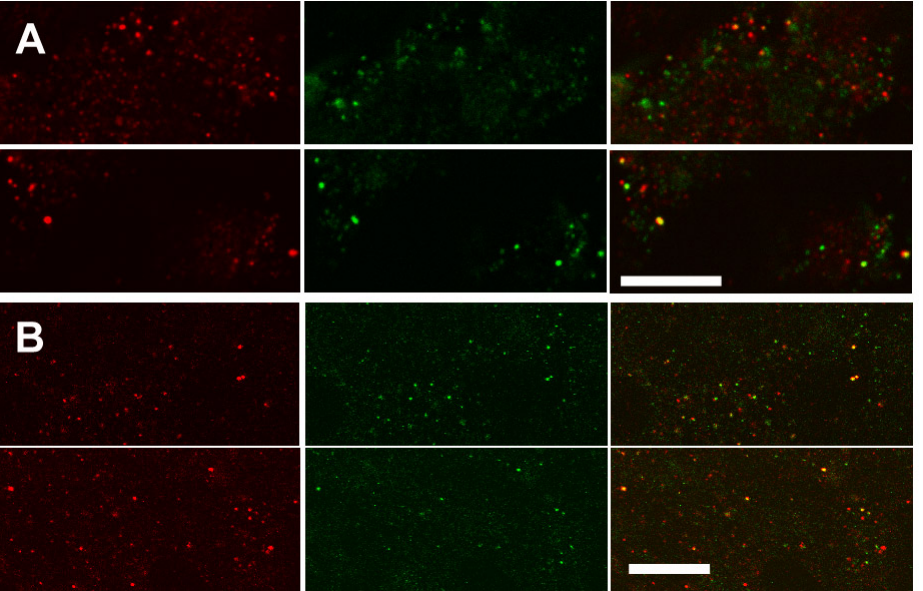
**Colocalization of CLIC1 with megalin and NaPi-II in MPTC cells**. MPTC cells were grown on collagen-coated filters, fixed, permeabilized, and stained for CLIC1 with the 9F5 monoclonal antibody (green, center column) and two markers of the apical endocytic/recycling compartment (red, left column). The right column in each set is a merged image generated from the red and green channels. **A. **Two separate fields from near the apical pole of the cells stained for megalin (red) and CLIC1 (green). **B. **Two separate field from near the apical pole of the cells stained for NaPi-II (red) and CLIC1 (green). The scale bars represent 10 microns in each set of images.

The distribution of CLIC1 in T84 cells suggests it is present in subapical intracellular vesicles. We have been unable to identify this compartment further in T84 cells, but it is clear that many polarized epithelial cells have abundant subapical vesicles which are involved in endocytosis and recycling. To investigate whether CLIC1 may be in this compartment, we studied a mouse kidney proximal tubule cell line, MPTC [28]. Kidney proximal tubule cells are well suited for this purpose since several well characterized proteins are known to transit through the endocytic/recycling compartment in these cells. Megalin, a multi-specific scavenging receptor for many components of the glomerular filtrate [25], and NaPi-II [26], the sodium-phosphate co-transporter, are two proteins which are known undergo endocytosis and, in the case of megalin, recycling to the plasma membrane in kidney proximal tubule cells. We grew MPTC cells on permeable supports under conditions to maximize differentiation. As in proximal tubule cells in intact kidney, these cells express abundant CLIC1 which shows a strongly apically polarized distribution (Fig. 12). Cells were fixed, permeabilized, and stained with rabbit polyclonal antisera to megalin or NaPi-II and costained with the 9F5 monoclonal antibody to CLIC1. Confocal images were collected from regions below the apical pole of the cells and are shown in figure 13. Clearly many of the punctuate structures that stain for megalin or NaPi-II also stain for CLIC1. However, there certainly are structures that stain for either megalin or NaPi-II but not for CLIC1, and structures that stain for CLIC1 but not for megalin or NaPi-II. Thus we conclude the CLIC1 compartment overlaps with the endocytic/recycling compartment, but is not identical to it.

## Discussion

In this paper, we have described the distribution of CLIC1 in normal adult mouse tissues and characterized the subcellular distribution of the protein in three distinctly different cell lines. Our data sheds new light on possible roles for CLIC1 in different tissues and cell types.

Prior northern blot data indicated that CLIC1 is widely expressed throughout the body, with particularly strong signals in muscle, liver, and kidney. However, northern blot data only reflect the presence of mRNA and do not necessarily perfectly correlate with the steady state level of encoded protein. Furthermore, northern blots using RNA isolated from whole organs are necessarily silent regarding heterogeneity of expression among the cells of a given organ or subcellular location within individual cells. Thus, the data presented here represents a significant new contribution to knowledge of CLIC1 expression patterns.

We found CLIC1 expressed in three distinct patterns. First, in several simple columnar epithelia, CLIC1 is expressed in an apically polarized pattern. Tissues showing this pattern include the epithelia of airways, intrahepatic biliary ducts, gall bladder, pancreatic duct, surface epithelia of the glandular stomach, small intestine and colon, in addition to the previously described renal proximal tubule [17]. A second pattern of staining is demonstrated by non-keratinizing stratified epithelia of the esophagus, and forestomach. In these tissues, the basal layer of cells express CLIC1 abundantly without clear subcellular specificity, although the prominence of staining of the periphery of the cells suggest CLIC1 may be present in plasma membrane. The epididymis, which is comprised of both columnar epithelial cells and basal cells, shows both patterns of staining. The basal cells stain throughout the organ, while only the tail of the epididymis exhibits staining of columnar cells. The third pattern of expression is found in the muscle cells of skeletal muscle. Here, CLIC1 is present throughout the cytoplasm.

Numerous tissues and cell types failed to stain for CLIC1. These cells may indeed express no CLIC1 at all, or they may express it at levels below the detection threshold of our staining method. These tissues include acinar secretory epithelia such as pancreatic acinar cells, subepithelial glands of lung and colon, and the submandibular salivary gland. Endocrine cells of the adrenal and the pancreatic islets failed to stain. Connective tissues surrounding each of the organs studied were consistently negative. Skin, spleen, smooth muscle of the gut, and the heart were all without significant staining for CLIC1.

These observations defy simple generalizations about CLIC1 expression. For example, the abundant expression in the rapidly dividing basal layer of the upper gastointestinal tract would be consistent with a role in cell cycle as previously proposed [18], but its absence from the equally rapidly dividing basal layer in skin epithelium or in generative nodules in spleen, rule out a generally required role in this process. Likewise, CLIC1 is abundant in skeletal muscle, but its absence in smooth and cardiac muscle rules out an absolute requirement for this protein in a muscle-specific function. The existence of other closely related CLIC family members complicates the analysis. There may indeed be a necessary function played by a CLIC in these processes, but a family member other than CLIC1 may carry out the role in tissues where CLIC1 is not prominently expressed. A consistent common thread does run through the epithelia which demonstrate apically polarized expression: these are all columnar epithelia which carry out transepithelial transport of salt and water and which have abundant apical membrane trafficking. Thus it is possible that CLIC1 may be functioning as a chloride channel in the apical plasma membrane or subapical intracellular vesicles in these cells.

Immunohistochemistry can be very useful for identifying cells and tissues expressing a particular antigen, but the limited resolution makes detailed subcellular localization difficult to determine. To define the membrane compartments in which CLIC1 may reside more precisely, we used immuofluorescence studies of three distinct cell lines: Panc1 cells, a poorly differentiated, non-polarized cell line derived from a human pancreatic cancer; T84 cells, a well-differentiated, highly polarized cell line derived from human colon cancer; and MPTC cells, an immortalized kidney proximal tubule cell line derived from a mouse transgenic for temperature-sensitive SV40 large T antigen. All three of these cell types express CLIC1 abundantly.

In the non-polarized cell line, PLP fixation followed by staining for CLIC1 reveals abundant CLIC1 in the cytoplasm in a punctate pattern. However, the bulk of the CLIC1 is soluble and not membrane-associated, as reflected both by differential centrifugation and digitonin extraction. Digitonin extraction removes the punctate cytoplasmic staining, indicating that this pattern was due to the abundant soluble CLIC1. Much of the digitonin-resistant CLIC1 colocalizes with plasma membrane markers, indicating that the primary site of membrane-inserted CLIC1 in these cells is the plasma membrane. The observation that the peripheral digitonin-resistant CLIC1 is solubilized by Triton X-100 supports the interpretation that CLIC1 in this distribution is present as a membrane protein and not as a component of the cytoskeleton. The variability of staining intensity among cells in culture is consistent with the variation with cell cycle of plasma membrane activity attributed to CLIC1 as previously reported [18]. Some of the digitonin-resistant CLIC1 colocalizes with the nucleus. However, it appears to be within the nucleus, not inserted into the nuclear envelope where one would expect CLIC1 if it were functioning as a nuclear membrane channel. Since the low concentration of digitonin used in these studies is not expected to permeabilize the nucleus [21], it remains possible that the residual nuclear CLIC1 observed following digitonin extraction is soluble rather than membrane-associated.

T84 cells showed a pattern of CLIC1 distribution quite different from that of Panc1 cells. These cells express CLIC1 in a distinctly apically polarized distribution, and this polarized expression is exaggerated following exposure to forskolin. In comparison to Panc1 cells, a larger fraction of CLIC1 is membrane-associated, both by differential centrifugation and by resistance to digitonin extraction. Furthermore, digitonin extraction does not dramatically alter the staining pattern of CLIC1 in T84 cells. Double-labeling experiments using a marker of the apical plasma membrane confirm that the polarized distribution of membrane-inserted CLIC1 is enhanced by treatment with forskolin and that the digitonin-resistent CLIC1 is not in the apical plasma membrane itself, but is concentrated immediately below it. The data indicate that the bulk of membrane inserted CLIC1 in T84 cells is in subapical membrane vesicles.

MPTC cells also show apically polarized distribution of CLIC1 which mirrors the distribution of CLIC1 in proximal tubule cells in intact kidneys. In these cells, the distribution of CLIC1 intersects with that of both megalin and NaPi-II, identifying at least a portion of the CLIC1-positive vesicles as components of the apical endocytic/recycling compartment. Endocytic and recycling vesicles are known to contain a chloride conductance which plays an important role in vesicular acidification which in turn is critical for orderly membrane traffic [1,2,29-31]. A different chloride channel, ClC5, has been implicated as a chloride channel of endocytic vesicles in proximal tubule [32,33]. Whether CLIC1 also plays a role as a chloride channel in this pathway remains to be determined. However, it does at least appear to be physically present in a fraction of the vesicles along this pathway.

## Conclusion

CLIC1 expression exhibits tissue- and cell type-specific patterns, both in the extent of expression and in subcellular distribution. In non-polarized cells such as basal epithelial cells of upper gastrointestinal tract, skeletal muscle, and cultured Panc1 cells, CLIC1 is expressed in a non-polarized distribution that appears to be throughout the cytoplasm. In the cultured cell model of non-polarized cells, the majority of the CLIC1 is present in a soluble form in the cytoplasm, while the membrane-inserted fraction of CLIC1 is primarily in the plasma membrane. In contrast, in some polarized epithelial cells, the expression of CLIC1 is strongly polarized to the apical domain. Using the cultured cells as a model, a larger fraction of the total CLIC1 is membrane-inserted; the membrane-inserted form of the protein is not primarily in the plasma membrane, but in subapical membranes vesicles which do not colocalize with markers of the endoplasmic reticulum, Golgi, or trans-Golgi network, but which do partially colocalize in with a subset of the apical endocytic/recycling compartment. Whether CLIC1 contributes to the chloride conductance and regulation of acidification of vesicles along this pathway remains to be determined.

## Methods

### Antibodies

Glutathione-S-transferase (GST)-CLIC1 fusion protein was produced in bacteria, purified to apparent homogeneity [12], and used to raise both a rabbit polyclonal antisera and a mouse monoclonal antibody, using the services of Cocalico, Inc., Reamstown, PA. The coding region of the CLIC1 component of the GST fusion protein is identical to cDNA sequences NM001288, BC095469, and BC064527 as well as the genomic sequence AF129756 in the NCBI data base. Thus the antigen is authentic human CLIC1. The polyclonal antiserum was affinity-purified using standard protocols [34], over a 6His-tagged CLIC1 which had been immobilized on Affigel 15 (Bio-Rad Laboratories, Richmond, CA). The resulting affinity-purified antisera, designated AP1089, was used at 1:1000 dilution for western blotting and at 1:100 dilution for cell staining. A polyclonal rabbit anti-β-galactosidase antibody which had been affinity-purified using identical methods was used as a negative control. The monoclonal antibody, designated 9F5, was generated in the form of ascites and was used at 1:1000 dilution for western blots and at 1:100 for cell staining.

Mouse monoclonal antibodies to Annexin II, Integrin α2, and Nucleoporin P62 were from the Organelle Detector Sampler Kit from BD-Transduction Laboratories, Franklin Lake, NJ. Monoclonal anti-Golgi 58 K protein and the monoclonal pan anti cytokeratin was obtained from Sigma, St. Louis, MO; monoclonal antibody to BiP was from Stressgen, Victoria BC, Canada; monoclonal anti-Lamp1 was from Calbiochem, San Diego, CA; sheep polyclonal antibody to TGN46 was from Serotec, Raleigh, NC. Rabbit polyclonal antibody to megalin [35] was provided by Dr. Marilyn G. Farquhar, University of California at San Diego, San Diego, CA. Rabbit polyclonal antibody to NaPi-II [36] was provided by Dr. Eleanor Lederer of the University of Louisville, Louisville, KY. Alexafluor 488- and 565-conjugated secondary antibodies were obtained from Invitrogen, Carlsbad, CA.

### Cell culture

Panc1 [37] and T84 [38] cells were obtained from the American Type Culture Collection, Manassas, VA. Panc1 cells were grown in DMEM (low glucose) with 10% fetal calf serum. T84 cells were grown in a 1:1 mixture of DMEM (low glucose) and Hamm's F12 with 5% newborn calf serum. All media was supplemented with 100 units/ml penicillin and 100 μg/ml streptomycin. For staining, Panc1 cells were plated on poly-L-lysine-coated glass cover slips and grown for at least two days prior to fixation. T84 cells were seeded at near-confluence on Collagen I-coated Millicel CM culture plate inserts (Millipore, Bedford, MA) and maintained in culture for 2–3 days after reaching apparent confluence. Vaccinia-driven expression of CLIC1 in HELA cells was as previously described [39].

MPTC cells [28] were derived from an otherwise normal mouse that is transgenic for a temperature sensitive SV40 large T antigen (Immortomouse, Charles River Labs, Wilmington MA). The cells were propagated at 32°C on collagen I coated Millicel CM tissue culture inserts in a 1:1 mixture of DMEM (low glucose):Hamm's F12 supplemented with 5 μg/ml insulin, 5 μg/ml transferrin, 10 ng/ml epithelial grown factor, 4 μg/ml dexamethasone, 50 μM L-ascorbic acid 2-phosphate, 10 units/ml γ-interferon, 15 mM HEPES, 1.2 mg/ml NaCO_3_, and 5% fetal bovine serum. For staining, the cells were grown to confluence in propagation medium, then switched to differentiation media and incubated for 2–3 days at 39°C. Differentiation medium for the basal chamber was the same as propagation medium except for the addition of 1 nM T3 and the omission of epithelial growth factor and interferon. Differentiation medium for the apical chamber was identical to basolateral differentiation medium except for the omission of fetal bovine serum. Care was taken so that the volume of medium in the apical chamber of a 0.6 cm^2 ^filter did not exceed 150 μl.

### Staining mouse tissue sections

Fresh organs from normal young adult mice were rinsed with cold phosphate buffered saline (PBS; 135 mM NaCl, 10 mM sodium phosphate pH 7.0), submerged in Tissue Tek embedding solution, and frozen in a dry ice/2-methyl butane bath. Five micron thick sections were obtained. Individual sections were thawed and immediately fixed with methanol at -20°C for 5 minutes. Endogenous peroxidase activity was inactivated by incubation with a 4:1 mixture of methanol and 3% hydrogen peroxide for 20 minutes at room temperature. Sections were blocked by incubation in PBS-fg (PBS with 5% goat serum, 0.02% fish gelatin) for 1 hour at room temperature, and then incubated with primary antibody overnight at 4°C in a humidified chamber. Sections were washed 4 times for 5 minutes each with PBS-fg and developed with horseradish peroxidase-labelled secondary antibody and diaminobenzadine, using the Envision+ System-HRP as instructed by the manufacturer (DAKO, Carpenteria, CA) and counterstained with Mayer's hematoxalin (Lille modification). Images were obtained using a Zeiss digital camera mounted on a Nikon Labphot microscope.

### Staining cultured cells

Cells were fixed in 2% PLP (2% (w/v) paraformaldehyde, 75 mM sodium phosphate, 75 mM lysine, 20 mM sodium periodate, pH 7.0) for 30 minutes at room temperature, blocked and permeabilized by incubation in PBS-fg with 0.05% (w/v) saponin for 30 minutes at room temperature. Primary antibodies were diluted in PBS-fg and incubated at 4°C overnight, washed 4 times with PBS-fg, incubated with combination of appropriate secondary antibodies (Alexafluor 488-conjugated goat anti mouse IgG and/or Alexafluor 568 conjugated goat anti rabbit IgG, each at 1:200 dilutions in PBS-fg) at room temperature for 1 hour, washed with PBS-fg 4 times and mounted in Vectorshield (Vector Laboratories, Burlingame, CA).

To label the apical surface of T84 cells, a confluent cell monolayer was rinsed with ice cold HEPES-buffered saline (HBS; 135 mM NaCl, 4 mM KCl, 1 mM CaCl_2_, 1 mM MgCl_2_, 1 mM Na_2_HPO_4_, 5 mM glucose, and 10 mM HEPES pH 7.4) and then incubated in HBS with 1 μg/ml FITC-conjugated wheat germ agglutinin (Sigma Chemical Co., St. Louis, MO) on ice for 10 minutes, rinsed four times with cold HBS, and then processed for staining as described above.

Fluorescently-labeled cells were imaged on a Bio-Rad laser-scanning confocal microscope with simultaneous collection of images in separate channels. To eliminate possibility of bleed-through of light between channels, red and green images were collected sequentially and absence of bleed-through was confirmed with single-stained specimens with identical image acquisition settings.

### Detergent extraction and cell fractionation

Cultured cells were rinsed with PBS and then incubated in 25 mM HEPES-KOH pH 7.0, 125 mM potassium acetate, 2.5 mM magnesium acetate, and 0.004% (w/v) digitonin for 2 minutes at room temperature. With T84 cells growing on filters, both apical and basolateral surfaces were exposed to digitonin. The cultures were then gently washed with PBS four times. Cells for immunofluorescence were immediately fixed in PLP and processed for staining as above. Cells to be fractionated which had been grown on a 100 mm cell culture dish were scraped into 1 ml of ice cold buffer A (PBS containing 1 mM ethylenediaminetetraacetic acid and 0.1 mM phenylmethylsulfonylfluoride) and homogenized by passage through a 25 gauge needle 10 times. The suspension was centrifuged at 2000 RPM for 10 minutes at 4°C in a table-top microcentrifuge. The supernatant was then centrifuged at 40,000 RPM (100,000 × G) at 4°C for 1 hour in a Beckman 70Ti rotor. The supernatant was taken as the soluble fraction. The pellet was dissolved in 0.25 ml of buffer A with 0.1% TritonX100. Protein concentration was determined using the BCA assay (Pierce Biochemicals, Rockford, IL).

For Triton X-100 extraction, cells were grown on poly-L-lysine coated cover slips for several days, rinsed with PBS and incubated in 10 mM HEPES pH 7.4, 100 mM NaCl, 5 mM MgCl_2_, 2.5 mM ethylene glycol-bis(β-aminoethyl ether)-N,N,N',N'-tetraacetic acid, 14 mM β-mercapto ethanol, 0.5% (v/v) Triton X-100, and 0.1 mM phenylmethylsulfanylfluoride at room temperature for 5 minutes [11,27]. The cells were gently rinsed with PBS, then fixed with PLP and processed for staining as above.

### Western blotting

Proteins were separated by SDS-PAGE and blotted to nitrocellulose. Blots were stained with Ponceau S to detect molecular mass standards. Blots to be probed with AP1089 were blocked in SuperBlock (Pierce Biochemicals); blots to be probed with 9F5 were blocked in Superblock supplemented with 1% nonfat dried milk. Signals were developed using horseradish peroxidase conjugated anti-rabbit or mouse IgG as appropriate and SuperSignal reagent (Pierce Biochemicals) followed by detection with X-ray film.

## List of abbreviations

GST: glutathione-S-transferase; PBS: phosphate buffered saline; HBS: HEPES buffered saline; PLP: paraformaldehyde, lysine, periodate fixative; WGA: wheat germ agglutinin.

## Authors' contributions

BU characterized the antibodies, performed immunostaining of culture cells and devised the digitonin extraction methods. JB performed immunostaining of tissue sections. PGW derived the mouse proximal tubule cell line. JCE conceived the study, was involved in collection of all the data, and drafted the manuscript. All authors read and approved the final manuscript.
